# A Model to Assess the Costs and Consequences of Changes in Diet and Nutrition From Potential Population-Wide Policies: The Microsimulation of Nutrition, Diabetes, and Cardiovascular Disease (MONDAC)

**DOI:** 10.5888/pcd22.250105

**Published:** 2025-10-09

**Authors:** Benjamin T. Allaire, Thomas Hoerger, Rainer Hilscher, Matthew B. Kaufmann, Simon Neuwahl, Lindsay M. Jaacks, Stephen Onufrak, Karen R. Siegel, Hui Shao, Michael Laxy, Ping Zhang

**Affiliations:** 1 RTI International, Research Triangle Park, North Carolina; 2 University of Minnesota, Health Policy and Management, Minneapolis; 3 University of Edinburgh, Roslin, Edinburgh, United Kingdom; 4 National Center for Chronic Disease Prevention and Health Promotion, Centers for Disease Control and Prevention, Atlanta, Georgia; 5 Hubert Department of Global Health, Rollins School of Public Health, Emory University, Atlanta, Georgia; 6 Emory Global Diabetes Research Center, Emory University, Atlanta, Georgia

## Abstract

**Introduction:**

The prevalence of diabetes continues to increase — more than 38 million people in the US now have diabetes and 84 million have prediabetes. Because many new cases of incident diabetes may be attributed to suboptimal dietary quality, novel programs and policies to encourage healthy eating choices represent promising population-level approaches to reduce the number of new cases of diabetes.

**Methods:**

To help estimate the potential impact of such programs and policies, we created the Microsimulation of Nutrition, Diabetes, and Cardiovascular Disease (MONDAC), a model to estimate the impact of simulated population-level dietary changes on downstream outcomes: body mass index, diabetes incidence, cardiovascular disease (CVD) incidence, all-cause mortality, quality-adjusted life years, direct medical costs, and cost-effectiveness.

**Results:**

We used 24-hour recall data from the National Health and Nutrition Examination Survey to categorize food and beverage consumption into 51 mutually exclusive categories to understand the effects of dietary changes. We simulated the energy intake and dietary quality effects that result from increasing, decreasing, or reallocating intake of these 51 food categories. Reductions in calories induce weight loss via an energy balance model. Weight loss and improvements in dietary quality drive annual reductions in diabetes and CVD risk. Mortality was modeled using a lifetable approach, and direct medical care costs were applied using estimates from the literature. We cross-validated MONDAC with existing models to assess reliability of estimates. We provide an example simulation for MONDAC, modeling a reduction in sugar-sweetened beverage consumption at the national level in the US.

**Conclusion:**

MONDAC provides a flexible approach to policy analysis to allow the user to simulate various food-related policies.

**Summary**

**What is already known on this topic?**

Dietary factors are major contributors to diabetes and cardiovascular disease risk. Simulation models can estimate health and economic impacts of dietary changes.

**What is added by this report?**

Microsimulation of Nutrition, Diabetes, and Cardiovascular Disease (MONDAC) is an innovative microsimulation model that integrates dietary changes, energy intake, weight, diabetes, and cardiovascular disease. It allows flexible policy analysis of food-related interventions on health outcomes and costs.

**What are the implications for public health practice?**

MONDAC provides a tool for policymakers to estimate long-term impacts of dietary policies. It can inform evidence-based decision making on food and nutrition interventions to prevent diabetes and cardiovascular disease.

## Introduction

The prevalence of diabetes continues to increase — more than 38 million people in the US now have diabetes and 84 million have prediabetes (1). Type 2 diabetes, which accounts for over 90% of diabetes cases, is largely preventable (2,3). Population-wide policies that lead to changes in major dietary risk factors in all people without diabetes could help decrease the risk of developing type 2 diabetes. However, governments and public health authorities require evidence-based policies and data to support implementation of food policy interventions.

Simulation models link epidemiologic and economic data and provide a tool to analyze and project the potential health and economic consequences of policies over time, therefore helping policymakers to determine the most effective policies and data to support policy implementation. Thus, a US-based simulation model that provides a comprehensive, convenient, and reliable way to generate the economic impact of food and nutrition policies could support evidence-based decision-making.

Food and nutrition policy simulation models have recently gained a foothold in the published literature (4). The models of Basu et al (5), Basu et al (6), and Mozaffarian et al (7) are microsimulation models estimating the risk of diabetes and cardiovascular disease (CVD) mortality caused by changes in nutrition policy in the Supplemental Nutrition Assistance Program. Long et al (8) and Lee et al (9) estimate the impact of national excise taxes on sugar-sweetened beverages (SSBs) on CVD risk.

In this article, we describe the development of a simulation model, Microsimulation of Nutrition, Diabetes, and Cardiovascular Disease (MONDAC). MONDAC improves on existing models by including both CVD and diabetes as outcomes and by using the latest risk equations available in the literature for diabetes and CVD risk. To better enable the simulation of a wider range of nutrition policies, MONDAC also adds to available models, such as Basu et al (10), by including the effect of changes in both total energy intake and dietary quality and the location where the food was obtained (eg, supermarket or grocery stores, convenience stores, restaurants) on diabetes and CVD outcomes. The primary purpose of MONDAC is to project long-term health and health-related economic effects of food and nutrition interventions and policies that aim to prevent diabetes and CVD.

## Methods

### Model structure

MONDAC is a microsimulation model that estimates how changes in quantity and quality of diet from potential population-wide food and nutrition policies might affect individual-level health outcomes over adults’ remaining lifetimes. The model simulates a person’s progression through disease modules for diabetes, CVD, and mortality. Food policies affect a person’s energy intake, dietary quality, and weight, which, in turn, affect the probability of disease progression. MONDAC estimates a person’s health care costs, health utility, and diabetes and CVD status each year, until the person dies or the scenario has ended. The model runs a food policy intervention versus the comparator of no intervention, and then compares these runs to calculate incremental costs, quality-adjusted life years (QALYs), and incremental cost-effectiveness ratio (ICER). ICERs provide a measurement of an additional cost per unit of health, in this case QALYs, to understand the value that a given intervention brings (11).

**Figure 1:**
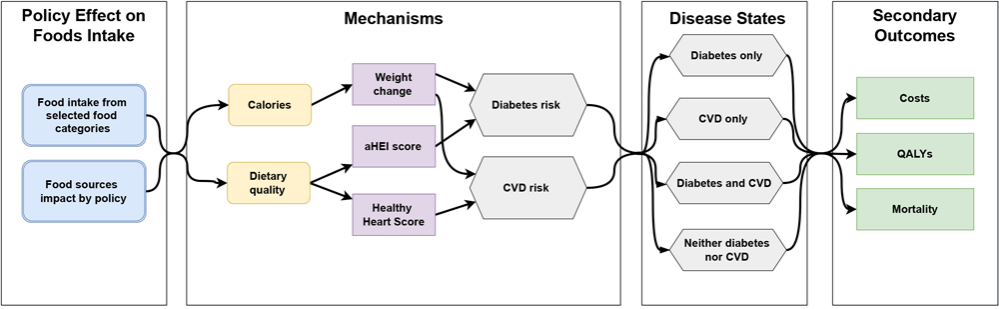
Microsimulation of Nutrition, Diabetes, and Cardiovascular Disease (MONDAC) model diagram for analyzing food intake and its effect on diabetes and cardiovascular disease (CVD) risk. Abbreviations: aHEI, Alternate Healthy Eating Index; QALYs, quality-adjusted life years.

Figure 1 provides an overview of the model, beginning with the anticipated effect of a food policy on intake of selected food categories (eg, SSBs, fruits, whole-grain breads) from various food sources (eg, supermarket, restaurant, convenience stores). It then follows the mechanisms by which changes in food intake impact diabetes and CVD risk and ends with the disease states and secondary outcomes. The model allows users to alter the diet of the people in the model and examine the impact on downstream outcomes: calories, dietary quality (Alternate Healthy Eating Index [aHEI]), weight status (body mass index [BMI]), diabetes incidence, CVD incidence, mortality, QALYs, and direct medical costs. Table 1 provides an overview of MONDAC’s modules and the sources for their inputs. All modeling was conducted in Python, version 3.9 (Python Software Foundation). MONDAC is available for use by Centers for Disease Control and Prevention (CDC) staff. The model can also be available for non-CDC researchers for nonprofit purposes. Researchers interested in using MONDAC should contact the senior CDC coauthor listed in the paper.

**Table 1:** Model Data Sets and Inputs for Microsimulation of Nutrition, Diabetes, and Cardiovascular Disease (MONDAC) Model^a^

Module	Input	Source
Food	Data set	National Health and Nutrition Examination Survey 2011–2012 and 2013–2014 (12)
	Dietary quality	Food Pattern Equivalent Database 2011–2012 and 2013–2014 (13)
	Food	What We Eat In America 2011–2012 and 2013–2014 (14)
Body weight	Body mass index equation	Hall et al (15)
Diabetes risk	Incidence equation	Alva et al (16)
	Risk reduction — body weight	Hamman et al (17)
	Risk reduction — diet quality	Ley et al (18)
Cardiovascular disease risk	Pooled cohort equation	Yadlowsky et al (19)
	Risk reduction — diet quality	Chiuve et al (20)
	First-year mortality	Koton et al (2014) (stroke mortality) (21); Colantonio et al (2017) (congenital heart disease mortality) (22)
Mortality	Life tables	Arias et al (23)
	Disease prevalence	Behavioral Risk Factor Surveillance System 2013 (24)
	Disease relative risks	Emerging Risk Factors Collaboration (25)
Health care costs	Diabetes costs	Shrestha et al (26)
	Cardiovascular disease costs	O’Sullivan et al (27); Shaw et al (28)
Quality of life	Quality-adjusted life year	Fu et al (29)

a For further information on the assumptions in MONDAC, an Appendix is available from the authors.

## Results

### Simulation population

The default baseline population for MONDAC is a nationally representative sample population that was generated based on adults from the 2011–2014 National Health and Nutrition Examination Survey (NHANES) population (30), which provides detailed data on the amount of specific food groups and nutrients consumed by people and demographic and clinical risk factor data. Each simulation samples 20,000 adults from NHANES with replacement and simulates the disease risks into the future, with existing diabetes and CVD cases included in the sampling. The sample is a fixed-entry cohort with no new entrants after initialization. To make the overall sample nationally representative, the model uses NHANES-provided dietary recall sample weights, which have been adjusted for nonresponse to the dietary recall survey and for the proportion of weekend and weekday interviews in the sample. MONDAC also allows the user to change the distribution of the population in terms of age, race, sex, or income. The user inputs their desired population size and then the percentage of the selected grouping (eg, 25% male). This allows the user to better simulate the actual population that a given policy is anticipated to affect.

### Policies considered

MONDAC provides a flexible platform to allow the user to simulate long-term health outcomes and costs of changes in the quantity and quality of dietary intake for various potential food-related policies. The user needs to supply the model with information on the policy costs, categories of food impacted by the policy, and sources of food (restaurants, supermarkets or grocery stores, convenience stores, or workplaces) impacted by the policy. MONDAC has 51 categories of foods (Table 2) to adjust and the user then chooses how calorie compensation occurs: 1) by replacing the calories with other specific foods (eg, replacing SSBs with fruit juice), 2) by replacing the calories from a specific food category with a proportional amount of calories from food categories already consumed by the individual, or 3) by replacing the calories with nothing (eg, replacing SSBs with 0 calories). MONDAC then calculates how the changes in intake of these food categories translate to changes in energy intake and dietary quality.

**Table 2:** Food Categories Available in the Microsimulation of Nutrition, Diabetes, and Cardiovascular Disease (MONDAC) Model^a^

Category	Description
1	Whole and reduced-fat milk
2	Low-fat and nonfat milk
3	Flavored milk
4	Yogurt and cottage cheese
5	Cheese
6	Other high-fat dairy
7	Non-lean meats
8	Poultry
9	Seafood
10	Eggs and omelets
11	Processed meats
12	Beans, peas, and soy
13	Nuts and seeds
14	Meat, vegetable, and starch mixed dishes
15	Vegetable and starch mixed dish
16	Pizza
17	Refined-grain sandwiches
18	Soups
19	Refined-grain pasta, breads, rice
20	Refined-grain high-sugar cold cereal (>21.2 g/100 g)
21	Refined-grain low-sugar cold cereal (<21.2 g/100 g)
22	Hot cereal
23	Whole-grain chips, pretzels, and snack mix
24	Refined-grain chips, pretzels, and snack mix
25	Low-sugar nutrition and cereal bars, trail mix (<21.2 g/100 g)
26	Baked desserts
27	Candy
28	Dairy desserts
29	Whole fruits
30	Dried fruits
31	Fruit juice
32	Non-raw vegetables (not including white potatoes)
33	Non-fried white potatoes
34	Fried white potatoes
35	Sugar-sweetened beverages
36	Diet drinks
37	Water
38	Reduced-calorie baked desserts
39	Reduced-fat or sugar dairy desserts
40	Raw vegetables
41	Reduced-fat yogurt and cottage cheese
42	Reduced-fat cheese
43	Whole-grain breads, rice, and pasta
44	Whole-grain high-sugar cold cereal (>21.2 g/100 g)
45	Whole-grain low-sugar cold cereal (<21.2 g/100 g)
46	Meat and vegetable mixed dishes
47	Meat and starch mixed dish
48	Meat mixed dish (no vegetable or starch)
49	Vegetable mixed dish (no meat or starch)
50	Starch mixed dish (no meat or vegetable)
51	High-sugar nutrition and cereal bars, trail mix (>21.2 g/100 g)

a Mutually exclusive categories were created from 24-hour recall data from the 2011–2014 National Health and Nutrition Examination Survey to categorize food and beverage consumption.

In addition to accommodating user-defined polices, MONDAC also includes 5 preset policies users can select as learning tools and examples. These example policies include preset values for policy costs, changes in intake of specific food categories, and food sources based on data in published literature. Finally, MONDAC also includes a feature whereby users can simulate the population effect of a change in overall energy intake or diet quality without specifying food categories or sources.

### Estimated policy effect on energy intake and dietary quality

MONDAC’s food module simulates changes to the diet of each person that could occur in response to a policy. Broadly, it estimates 2 quantities in response to user-inputted changes to foods consumed, namely the change in energy intake (calories) and the change in diet quality (Figure 1). The user may derive these changes in food and nutrition intake from the published literature or from their own data collection in response to a program, practice, or policy, with the acknowledgment that the changes simulated by MONDAC would be hypothetical in nature. The change in calories is then relayed to the weight module, which simulates weight change. Diet quality impacts are captured through the aHEI for diabetes risk and through the Healthy Heart Score — Dietary Component (HHS-DC) for CVD risk. The aHEI is an 11-dimension dietary quality index based on a combination of food and nutrient variables that have established relationships with chronic disease risk (31). The HHS-DC was created by Chiuve et al (20) and predicts CVD risk based on demographic and lifestyle factors, including BMI, dietary quality, exercise, and other health behaviors, such as smoking and drinking, which have been selected from NHANES. We linked the combined 2-day 24-hour recall data to the Food Pattern Equivalent Database (13) to calculate changes in overall aHEI and HHS-DC scores. The 24-hour dietary recall lists the grams of each food consumed by respondents in the 24 hours before the survey. Foods were identified by Food and Nutrient Database for Dietary Studies 2013–2014 (32) food codes. The foods fall into 153 categories, which we condensed into 51 categories (Table 2). To create our modified category list, we started by finding which What We Eat In America (WWEIA) (14) categories were the most significant sources of calories based on NHANES data. We also focused on categories that were targeted in the Food Service Guidelines for Federal Facilities (33) and the 2015–2020 Dietary Guidelines for Americans (40). Several WWEIA categories were aggregated, while others were disaggregated. For example, we combined the WWEIA categories for soft drinks, fruit drinks, and sports and energy drinks into a single category for SSBs. Likewise, we combined all WWEIA categories corresponding to pasta, breads, and rice into 1 category and then disaggregated this category into whole grains and refined grains. Some categories were excluded either because they were not relevant for adults (eg, formula, baby water) or because they are unlikely to be targeted by food policies (eg, protein and nutritional powders, smoothies, grain drinks).

Because the MONDAC food module simulates the potential effect of food policies that may be implemented in the community (restaurants, grocery stores, and convenience stores) or in workplaces, the source (place obtained) of consumed food is an important factor. We incorporated food source as an input variable so that the user can independently model the effect of policies on food from restaurants, workplaces, supermarkets or grocery stores, and convenience stores. Sources were categorized into these 4 categories based on judgment by the authors from 28 original NHANES food source categories.

### Body weight module

The body weight module estimates reductions in weight in response to reductions in energy intake using the model specified in the National Institute of Diabetes and Digestive and Kidney Diseases (NIDDK) model (15). The NIDDK model has been validated by using repeated measurements over a 2-year study, Comprehensive Assessment of Long-Term Effects of Reducing Intake of Energy (34). (An Appendix for the equations used in the weight module is available from the authors.) Because dietary changes that result from interventions may not be permanent, the bounce-back effect was applied similarly in the weight module as in the food module; weight reduction was assumed to be halved after 1 year and set to zero after 2 years.

### Diabetes risk

We estimated diabetes incidence by using the equations from Alva et al (16) using variables for age, race, sex, BMI, smoking status, cholesterol levels, parental history of diabetes, and blood pressure at baseline. We modified each person’s annual diabetes risk with risk reductions based on changes in body weight and diet quality (aHEI) (Figure 1). We assumed that these effects act independently on the person. The diabetes risk reduction from body weight comes from Hamman et al (17), which was an analysis of the lifestyle intervention of the Diabetes Prevention Program (DPP). The diabetes risk reduction from diet quality comes from Ley et al (18), which used the 2010 version of the aHEI. When the user-entered policy changes the diets of the people in the model, the aHEI score will increase or decrease. We used the estimates from Ley et al (18) to quantify the diabetes risk reduction associated with changes in total aHEI while controlling for self-reported baseline BMI.

### CVD risk

To model CVD risk, we used the Yadlowsky et al (19) update to the American Heart Association (AHA) Pooled Cohort Equations, originally from Goff et al (35), to predict background CVD risk based on age, sex, race, blood pressure, diabetes, cholesterol, and smoking status and the dietary quality components of the HHS-DC to provide risk reductions in response to policy changes (31).

Hard atherosclerotic cardiovascular disease (ASCVD) is defined by 4 events: nonfatal myocardial infarction, fatal coronary heart disease, fatal stroke, and nonfatal stroke. Because the outcome of the Pooled Cohort Equations is hard ASCVD, which includes both fatal and nonfatal CVD, a portion of those who develop CVD will die in the year of their first CVD event rather than moving into the population with CVD. To capture this group, we calculated mortality in the first year for the 2 outcomes that make up hard ASCVD: stroke and coronary heart disease.

### Mortality

We took a life table approach to mortality in the model by linking annual probabilities of death to different age groups. We obtained all-cause mortality for the entire population from the National Vital Statistics Reports (23). To estimate the mortality of 4 populations — diabetes only, CVD only, diabetes and CVD, and neither diabetes nor CVD — we extracted relative risks for all-cause mortality for each population from the Emerging Risk Factors Collaboration (25), which examined all-cause mortality for each of our subpopulations of interest.

### Costs and utility

Annual diabetes-attributable direct medical cost estimates were ascertained from Shrestha et al (26), which estimated longitudinal costs from the Truven MarketScan population (private insurance population). We separated CVD costs into 2 categories: first-year CVD costs and subsequent-year costs. First-year CVD costs were used from O’Sullivan et al (27). For costs beyond the first year, we relied on Shaw et al (28), who estimated 10-year medical costs for previously asymptomatic people. To calculate QALYs for cost-effectiveness analyses, we combined life expectancy with utility differences reported by Fu et al (29), who used nationally representative data from the Medical Expenditure Panel Survey for 2001 and 2003. MONDAC relies on user-supplied policy costs for each scenario. Model outcomes are discounted at a default 3% rate.

### Probabilistic sensitivity analysis

We accounted for parameter uncertainty by using probabilistic sensitivity analysis (PSA). PSA involves running the model repeated times with all model input parameters varied simultaneously within a priori–defined probability distributions. Using PSA via a Monte Carlo process allowed us to obtain both error bounds and confidence intervals on our outputs and cost-effectiveness acceptability curves (CEACs). CEACs summarize the impact of uncertainty on cost-effectiveness results with respect to different possible willingness-to-pay thresholds. CEACs are drawn as an alternative to constructing confidence intervals for the ICERs (36). We consulted Briggs et al (11) to select the most appropriate distributions to vary the parameters.

### Example: applying MONDAC to examine the long-term health and economic impact of reduction in consumption of SSBs

To provide an example of the potential use of MONDAC, we applied the model to SSB consumption. Overall SSB consumption has declined modestly among people in the US from 2003 to 2018 (37,38). Yet the implications of the decrease in SSB consumption are not well understood. We used MONDAC to understand the health implications resulting from a permanent, modest reduction in the consumption of SSBs.

We followed Long et al (8) and modeled a 20% reduction in SSB consumption in a simulated nationally representative population and 9 different subpopulations by age, sex, and income. Consistent with Long et al (8), the calories foregone by reducing SSB consumption will be replaced by 19% fruit juice. For example, if the person consumed 100 calories of SSBs per day, they would forgo 20 SSB calories (ie, a 20% reduction) that would be replaced by 3.8 (19% of 20) calories of fruit juice. The cost of implementing the policy change, which leads to the 20% lower SSB consumption, was assumed to be $0.17 per person in the first year and $0.14 in subsequent years. These cost estimates were obtained from previous studies and considered government implementation costs and compliance costs (8). We examined the impact in 100,000 people over 10 years using a 3% annual discount rate. We conducted a PSA to reflect the level of uncertainty of interest and generated CEAC curves.

Table 3 displays the incremental differences between a simulated population without the reduction and one with the reduction. Nationally, we estimated that a permanent 20% reduction in SSBs in the US would result in an aHEI increase of 0.25 and a calorie-per-day decrease of 35. These dietary changes translate into an average BMI reduction of 0.71 among SSB drinkers. The 20% reduction in SSB consumption would result in 137 fewer CVD cases, 1,387 fewer diabetes cases, and 60 fewer deaths among the 100,000 simulated study population over 10 years.

**Table 3:** Changes in aHEI, Energy Intake, BMI, Diabetes, CVD, and Deaths From a Permanent 20% Reduction in Consumption of SSBs With 19% Fruit Juice Compensation^a^ for a Population of 100,000^b^

Subgroup	Changes in health effects at the end of the 10-year simulation						SSB consumption prevalence
	aHEI score^c^	Energy intake^c^^,^ d	BMI^c^	Cumulative incidence — CVD	Cumulative incidence — diabetes	Deaths	
Nationally representative baseline	0.25	−34.81	−0.71	−137	−1,387	60	60.4%
Age 18–34 y	0.25	−40.82	−0.78	−37	−1,327	7	72.8%
Age 35–49 y	0.23	−38.25	−0.79	−90	−1,967	60	60.8%
Age 50–64 y	0.24	−28.24	−0.63	−156	−1,473	100	55.7%
Age ≥65 y	0.29	−21.17	−0.52	−150	−553	97	46.8%
Women	0.27	−28.23	−0.70	−66	−1,240	54	55.3%
Men	0.22	−39.04	−0.70	−180	−1,357	87	65.4%
Income <130% FPL	0.25	−42.57	−0.89	−203	−1,837	134	71.9%
Income 131%–199% FPL	0.25	−35.32	−0.76	−164	−1,677	83	67.4%
Income 200%–299% FPL	0.24	−36.94	−0.75	−107	−1,543	47	60.5%
Income ≥300% FPL	0.23	−29.20	−0.59	−110	−983	64	53.1%

Abbreviations: aHEI, Alternate Healthy Eating Index; BMI, body mass index; CVD, cardiovascular disease; FPL, federal poverty level; SSB, sugar-sweetened beverage.

a Nineteen percent of SSB calories that were reduced were compensated with calories from 100% fruit juices.

b Changes reflect differences between intervention population and nonintervention population. Starting population was set to 100,000 and the number of SSB drinkers in the nationally representative baseline population was 60,357 (SSB prevalence = 60.4%). Outcomes are reflective of SSB drinkers only.

c Per person statistics are presented.

d Energy intake presented in kCal per day per person.

For our subgroup analyses, generally, the higher the likelihood of SSB consumption, the more health impacts were associated with reductions in consumption. One exception was age groupings, where energy intake reduction is largest for those aged 18 to 34 years (−41 kCal/d per person) yet the highest reduction in diabetes incidence occurs for those aged 35 to 49 years, the result of diabetes onset later in life. Men experience much larger impacts than women with larger reductions in diabetes, and there is a steep income gradient associated with health impacts.

All subgroups were cost saving (Table 4). The average QALY increase is 0.0068 (95% CI, 0.0045–0.0099), and direct medical costs per person decrease $683 (95% CI, −860 to −576). The biggest health and cost impacts are felt by older age groups and among those with income below 130% of federal poverty level.

**Table 4:** Change in Costs, QALYs Gained, and ICER From a Permanent 20% Reduction in SSBs and 19% Fruit Juice Compensation^a^, Microsimulation of Nutrition, Diabetes, and Cardiovascular Disease (MONDAC) Model

Characteristic	Mean costs per person, $, 2024	Mean QALYs per person (95% CI)^b^	ICER ($ per QALY)
Nationally representative baseline	−$683 (−860 to −576)	0.0068 (0.0046 to 0.0132)	Cost savings
Age 18–34 y	−$510 (−609 to −398)	0.0032 (0.0016 to 0.0061)	Cost savings
Age 35–49 y	−$874 (−1,180 to −829)	0.0075 (0.0049 to 0.0141)	Cost savings
Age 50–64 y	−$827 (−1,053 to −692)	0.0097 (0.0061 to 0.0147)	Cost savings
Age ≥65 y	−$407 (−561 to −306)	0.0079 (0.0056 to 0.0138)	Cost savings
Women	−$655 (−875 to −578)	0.0066 (0.0035 to 0.0112)	Cost savings
Men	−$674 (−894 to −535)	0.0071 (0.0055 to 0.0125)	Cost savings
Income <130% FPL	−$740 (−1,060 to −693)	0.0102 (0.0049 to 0.0125)	Cost savings
Income 131%–199% FPL	−$745 (−988 to −628)	0.0079 (0.0051 to 0.0154)	Cost savings
Income 200%–299% FPL	−$746 (−975 to −660)	0.0063 (0.0047 to 0.0136)	Cost savings
Income >300% FPL	−$552 (−764 to −464)	0.0068 (0.0032 to 0.0108)	Cost savings

Abbreviations: ICER, incremental cost-effectiveness ratio; QALY, quality-adjusted life year; SSB, sugar-sweetened beverage.

a Changes reflect differences between intervention population and nonintervention population. Starting population was set to 100,000 and the number of SSB drinkers in the nationally representative baseline population was 60,357 (SSB prevalence = 60.4%). The proportion of SSB drinkers in other subgroups ranged from about 47% to 73% (Table 3).

b 95% CIs resulting from probabilistic sensitivity analyses (the 25th and 975th results).

**Figure 2:**
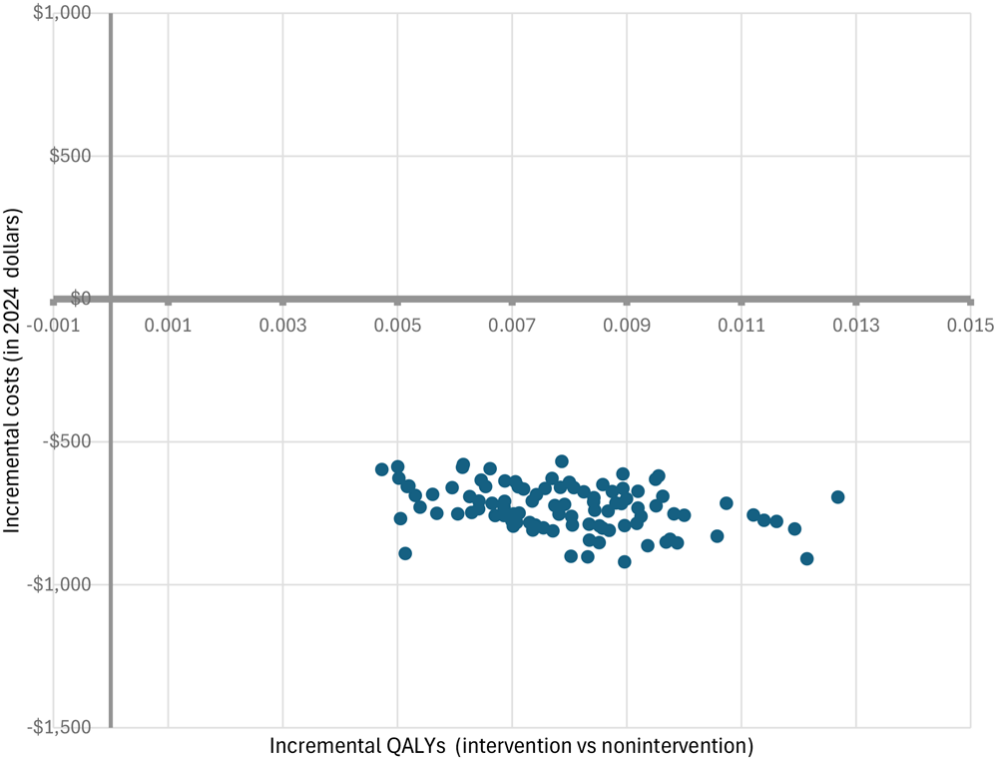
Incremental cost-effectiveness ratio (ICER) plot for policy to reduce consumption of sugar-sweetened beverages (SSBs). The axes for this figure are the incremental differences in quality-adjusted life years (QALYs) on the X axis and the costs on the Y axis. Positive differences represent cases where the intervention group is higher than the nonintervention group, while negative cases represent areas where the intervention group is lower than the nonintervention group.

Figure 2 presents the ICER plot from the PSA. All ICER points locate in the fourth quadrant of the ICER plane, implying that there was little uncertainty that the anticipated 20% reduction in SSB consumption from this policy change would be cost saving.

## Discussion

Dietary intake is a well-established risk factor for both diabetes and CVD. To assist policymakers, we designed the MONDAC model to enable the testing of a diverse array of dietary changes in terms of their impacts on diabetes and CVD in the US. As a preliminary example, we demonstrate that a policy that would reduce the consumption of SSBs by 20% could reduce incident diabetes and CVD cases by 1,387 and 137 per 100,000 population, respectively, over a 10-year period, resulting in direct medical cost savings. The model’s user-friendly interface and input flexibility enables users to tailor the dietary modifications and the types of settings in which they occur to test and provide scientific evidence to support implementation of food and nutrition policies among various populations in the US. MONDAC can handle various calorie compensation options, which is a unique and important contribution of this model to the scientific literature that will allow users to understand how various assumptions for compensation affect the impact of the policy. Further, unlike previous food policy simulation models that only account for changes in energy intake, MONDAC also incorporates the effect of improvements in diet quality on diabetes and CVD outcomes.

### Limitations

MONDAC has several limitations. First, it only includes health care and policy costs and does not include informal health care costs, or non–health care sector costs such as productivity, consumption, and social services. Thus, it underestimates policy cost saving. However, the cost data in the model may be nonrepresentative, such as using managed care cost data for medical costs, as managed care enrollees may have higher health care usage leading to an overestimate of cost savings. Second, we predict CVD risk based on the Pooled Cohort Equation and apply reductions from improvements in aHEI from the HHS-DC. Although there is substantial overlap between the outcomes of the HHS-DC CVD risk equation (nonfatal myocardial infarction [MI], fatal coronary heart disease, and ischemic stroke) and the Pooled Cohort CVD risk equation (hard ASCVD: nonfatal MI, fatal coronary heart disease, and fatal and nonfatal stroke), they are not perfectly aligned, which may result in misspecification of disease outcomes. Users may disable the HHS-DC risk adjustment when configuring their simulation if they are concerned that the differences between these 2 scores are unacceptable. Third, despite efforts to account for those with multimorbidity, the model may overestimate costs in cases where individuals have both diabetes and CVD. Fourth, we used overall aHEI and HHS-DC diet quality scores to predict change in diabetes and CVD risk, respectively, rather than focusing on particular component scores of each. There may be some elements of diet quality (corresponding to aHEI or HHS-DC component scores) that may be more important determinants of diabetes or CVD risk than other determinants. For example, the effects of changes in intake of added sugars may be different from those of changes in intake of proteins or sodium. However, this is a limitation of the available research in this area. Fifth, we are limited in several ways by NHANES dietary data. The data are from 2013 and 2014, and dietary practices may have changed since the creation of the model. However, the age of the data analyses suggests that changes in dietary patterns may be small. For example, the Healthy Eating Index changed 1.1 points, from 56.6 to 57.7, over the 10-year period from 2005–2006 to 2015–2016 (39). Another issue is that we were unable to estimate usual intake of food categories because of the difficulty of doing so for each person from all the specific food sources. Therefore, the 2-day average estimates of dietary intake for each food category used in the model may not reflect actual long-term intake of each food category, particularly for foods that are less frequently consumed, such as fish. Also, the food source categories we established may not correspond exactly with targets for policies because of the way they are categorized in NHANES data. Because of limited empirical evidence, anticipated effect of the policy on calorie consumption or diet quality may be difficult to know with certainty; thus, users should be cautious when interpreting impacts from MONDAC. Finally, NHANES data may also produce small cells for certain combinations of foods, sources, and demographic subgroups that limit the representativeness of estimates and suitability of error estimates.

### Conclusion

MONDAC offers a practical and flexible tool for assessing the health and health-related economic impacts of various food and nutrition policies. The model integrates key data on dietary behaviors, disease risks, and health care costs, allowing users to simulate various dietary interventions and estimate their potential long-term effects on diabetes and CVD outcomes. Further, the effects of policies on dietary intake and their effect on diabetes and CVD risk can be updated in the model by users as new evidence emerges in the literature. By considering variations in dietary quality and calorie compensation, MONDAC aids in the evaluation of policy scenarios, such as the reduction in SSB consumption. Although all simulation models have certain limitations, MONDAC provides a useful framework for supporting evidence-based decisions in public health. Ongoing efforts will aim to further validate and refine the model, with the goal of enhancing its application to a broader range of dietary interventions.
